# NG-meta-profiler: fast processing of metagenomes using NGLess, a domain-specific language

**DOI:** 10.1186/s40168-019-0684-8

**Published:** 2019-06-03

**Authors:** Luis Pedro Coelho, Renato Alves, Paulo Monteiro, Jaime Huerta-Cepas, Ana Teresa Freitas, Peer Bork

**Affiliations:** 1 Structural and Computational Biology Unit, European Molecular Biology Laboratory, Heidelberg, Germany; 2 Institute of Science and Technology for Brain-Inspired Intelligence, Fudan University, Shanghai, China; 3 Key Laboratory of Computational Neuroscience and Brain-Inspired Intelligence (Fudan University), Ministry of Education, Shanghai, China; 4 Collaboration for joint PhD degree between EMBL and Heidelberg University, Faculty of Biosciences, Heidelberg, Germany; 5 INESC-ID, Instituto Superior Técnico, University of Lisbon, Lisbon, Portugal; 6 Centro de Biotecnología y Genómica de Plantas, Universidad Politécnica de Madrid (UPM), Instituto Nacional de Investigación y Tecnología Agraria y Alimentaria (INIA), Madrid, Spain; 7 Max Delbrück Centre for Molecular Medicine, Berlin, Germany; 8 Molecular Medicine Partnership Unit, University of Heidelberg and European Molecular Biology Laboratory, Heidelberg, Germany; 9 Department of Bioinformatics, Biocenter, University of Würzburg, Würzburg, Germany

**Keywords:** Metagenomics, Next-generation sequencing, Domain-specific language

## Abstract

**Background:**

Shotgun metagenomes contain a sample of all the genomic material in an environment, allowing for the characterization of a microbial community. In order to understand these communities, bioinformatics methods are crucial. A common first step in processing metagenomes is to compute abundance estimates of different taxonomic or functional groups from the raw sequencing data.

Given the breadth of the field, computational solutions need to be flexible and extensible, enabling the combination of different tools into a larger pipeline.

**Results:**

We present NGLess and *NG-meta-profiler*. NGLess is a domain specific language for describing next-generation sequence processing pipelines. It was developed with the goal of enabling user-friendly computational reproducibility. It provides built-in support for many common operations on sequencing data and is extensible with external tools with configuration files.

Using this framework, we developed *NG-meta-profiler*, a fast profiler for metagenomes which performs sequence preprocessing, mapping to bundled databases, filtering of the mapping results, and profiling (taxonomic and functional). It is significantly faster than either MOCAT2 or htseq-count and (as it builds on NGLess) its results are perfectly reproducible.

**Conclusions:**

*NG-meta-profiler* is a high-performance solution for metagenomics processing built on NGLess. It can be used as-is to execute standard analyses or serve as the starting point for customization in a perfectly reproducible fashion.

NGLess and NG-meta-profiler are open source software (under the liberal MIT license) and can be downloaded from https://ngless.embl.de or installed through bioconda.

**Electronic supplementary material:**

The online version of this article (10.1186/s40168-019-0684-8) contains supplementary material, which is available to authorized users.

## Background

Over the last decade, metagenomics has increasingly been applied to the study of microbial communities. Most work has focused on human-associated habitats [1] with a particular emphasis on the human gut microbiome [2, 3]. However, the same methodologies have been used for studying other host-associated microbiota [4–6] or the marine microbiome [7]. Due to its size and complexity, several computational approaches have been proposed to handle these data, including bioinformatic pipelines combining different tools and approaches [8–11].

A typical metagenomics processing workflow can be divided into two distinct phases: in the first phase, raw data is processed (often using prebuilt reference databases) to generate a table of feature abundances (a profile). These features can be either taxonomic or functional annotations. Secondly, these profiles are analyzed (often in regards to relevant metadata) using statistical methods and packages such as phyloseq [12], vegan [13], or LEfSe [14]. In this work, we are focused on the first phase: namely obtaining functional and taxonomic profiles from raw metagenomic reads.

To this end, we present *NG-meta-profiler*, a collection of pre-configured pipelines based on the domain-specific language NGLess (Next Generation Language for less effortful analysis). Although *NG-meta-profiler* can be used as a standalone tool, the syntax and semantics of NGLess have been designed to be simple and human readable, allowing users to read or create their own pipelines, even without deep bioinformatics and programming knowledge. In other scientific contexts, domain-specific languages have been empirically found to increase productivity and user satisfaction [15, 16]. At the same time, NGLess is designed to enable perfect reproducibility of the computational process, an increasingly important concern [17–19].

## Implementation

### NGLess, a domain-specific language for NGS processing

NGLess is a domain-specific language which was designed specifically for next-generation sequence (NGS) processing. NGLess was initially designed by analyzing the intended use cases with potential users. NGLess is an imperative programming language, given that most bioinformaticians are familiar with languages using this paradigm [20]. The result contains types for concepts that were found to be important in the problem domain, such as *ShortRead* (representing a single short read) or *ShortReadSet* (representing a set of, possibly paired-end, short reads). Unlike most programming languages, values of these types are often represented by files on disk and not values in memory due to their large size.

We also conducted a user study (with a sample of convenience, with participants recruited locally and through social media) on an earlier version of the language, which revealed some issues in those earlier versions. Experience from tutorials on using the language was also used to simplify some aspects of the language. For example, in an earlier version of NGLess, there existed a construct to preprocess short-reads using pass-by-reference, where the argument to the *preprocess* function was modified. We found that changing this to pass-by-value led to fewer misunderstandings. Thus, the language now uses exclusively pass-by-value semantics. Another area where progress benefited from user input on beta versions is the quality of the error messages. Even when usage reports were due to user errors or malformed input files, we took this opportunity to improve the error reporting in NGLess to help the users diagnose and debug the underlying issue.

The built-in knowledge of the sequence processing domain allows for best-practices to be automatic. For example, our tool always collects quality control statistics without the user having to specify it as an additional computational step.

Domain knowledge enables the interpreter to perform computations more efficiently. For example, even though users write their pipeline script in a purely linear fashion, the interpreter can automatically detect when parallelization opportunities are available and take advantage of multiple processors in shared memory machines. In particular, loops are automatically parallelized, and format conversions (e.g., compression/decompression) use separate threads. When relying on external tools, NGLess can automatically detect possibilities to avoid intermediate files, while handling all format conversions internally.

Finally, error detection and reporting are significantly improved by having the tool be semantically aware of its goals. Given that debugging consumes a significant fraction of the time invested in developing computational pipelines, fast error detection can speed up the overall project. For example, it is possible to check whether inputs are readable and outputs are writable prior to starting interpretation. This benefits the user whenever they have made a mistake as errors are detected and reported immediately.

While introducing a novel language implies that the user needs to learn a new tool, the language is designed to be easily understandable to scientists familiar with the field. Alternatively, a Python interface to NGLess allows users familiar with that programming language to access NGLess functionality. Similarly, *NG-meta-profiler* is a command line tool that can be used directly without knowledge of NGLess.

In version 1.0, NGLess implements the following tasks: (1) preprocessing, (2) assembly, (3) open-reading frame (ORF) finding, (4) mapping to sequence databases, (5) filtering of mapping results, (6) profiling (up-to-date taxonomic and functional profiling databases are provided), and (7) summary plots. See Additional file 4: Text S1 and the online manual for a full description of the language, including the complete grammar in extended Back-Naur notation as well as documentation for all the built-in types, functions, and modules.

### NG-meta-profiler: a fast metagenomics profiler

Using the NGLess framework, we have developed *NG-meta-profiler*, a collection of pipelines for taxonomic and functional profiling of metagenomes. These standard analyses can be run with a single command. However, they can also serve as a starting point for customization by the user, including extending them with novel tools.

These pipelines are defined in the NGLess language, and currently, there are workflows available for human, mouse, pig, and dog gut as well as marine metagenomes. In the future, we plan to continually update the resources (annotated gene catalogs and other databases) for these environments, as well as providing resources for other environments.

**Fig. 1:**
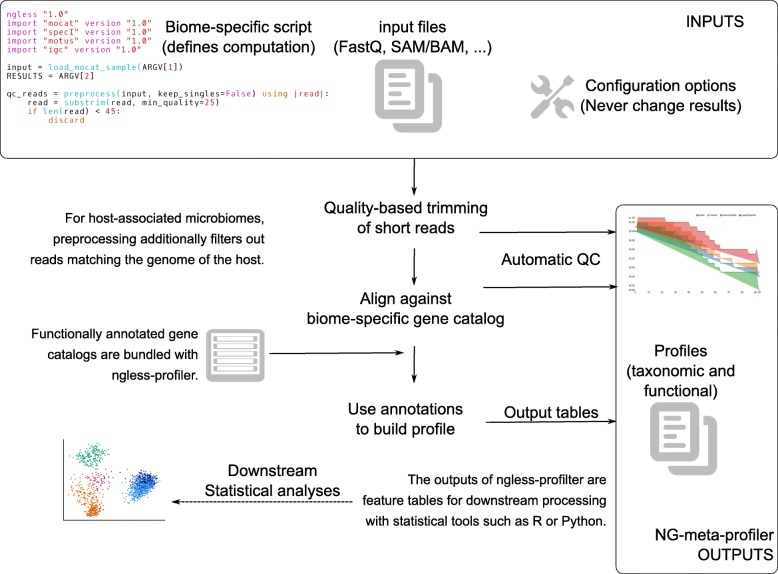
Schematic illustration of *NG-meta-profiler*

At a high level, NG-meta-profiler, performs the following operations: (1) preprocess the reads (performing quality-based trimming and filtering of short reads and, if appropriate, discarding reads that align to the host), (2) map the reads against a predefined gene catalog selected for that biome, and (3) use the predefined gene annotations to build a profile (see Fig. 1).

For preprocessing the short-reads, we use built-in NGLess constructs to perform quality based trimming and filtering of short reads. In NG-meta-profiler, we use the *substrim* function to trim reads. This function is built into NGLess and trims the sequence to the longest substring of its input such that all bases have at least the requested quality (the same approach was initially introduced by SolexaQA [21]). We keep only reads above quality value 25, which is the central point of the recommended range (20 to 30) in a previous study of the effects of read trimming [22]. Finally, we discard reads shorter than 45 base pairs as shorter reads are significantly more likely to align in multiple locations. These defaults can easily be changed by the user.

After preprocessing, it is necessary to remove reads that match the human genome. For this operation, we will first align the reads against the hg19 built-in reference.

As the default mapper is a local aligner (bwa-mem), it aligns reads even when only a short substring maps. To remove these spurious alignments, we remove very short alignments, namely those below 45 bp (see Additional file 1: Figure S1).

To obtain functional profiles from metagenomes, we use pre-classified gene catalogs as references, an approach that was previously shown to outperform per-read classification [8, 9, 23, 24].

#### Bundled databases and modules

As part of the first release of NG-meta-profiler, we bundle several genomes (including human and mouse) as well as gene catalogs for the human gut microbiome [25], the marine microbiome [7], and three non-human mammal microbiomes (pig [5], dog [6], and mouse [4]). These gene catalogs have been functionally annotated with eggnog-mapper [23] so that functional profiles can be generated by NGLess (see Table 1). In the current version, *NG-meta-profiler* can produce eggNOG orthologous group [26], KEGG orthologous groups (KO) [27], SEED [28], and BiGG [29] abundance profiles. Additionally, genomes of several organisms (see Additional file 4: Text S1) are also provided. These are used for filtering out host reads in host-associated metagenomes in *NG-meta-profiler*.

**Table 1:** Gene catalogs bundled with NG-meta-profiler

Database	Size (million genes)	Comment
igc	9.9	Integrated gene catalog for the human gut [25]
om-rgc	40	Ocean microbial gene catalog [7]
mouse-gut	2.6	Gene catalog of the mouse gut [4]
pig-gut	7.7	Gene catalog of the pig gut [4]
dog-gut	1.2	Gene catalog of the dog gut [6]

All of these resources are automatically downloaded by NGLess the first time they are used so that the initial download is small (currently 15 MiB) and each user only downloads those resources they effectively use. In addition to these, the user can provide external references by specifying the file paths to the resources on disk or writing an external module (which can be defined using a text file).

Several operations in NGLess are performed with bundled software. De novo assembly is performed using MEGAHIT [30], which has been found to perform well for metagenomics [31, 32]. Open reading frame (ORF) finding is performed with Prodigal [33]. By default, mapping is performed using bwa [34], but minimap2 [35] is also provided and SOAPaligner [36] can be used. Additional built-in modules provide extra functionality as shown in Table 2.

**Table 2:** NGLess built-in modules that add extra functionality

Module name	Comment
parallel	Process multiple samples in parallel
mocat	Compatibility with MOCAT/MOCAT2 [8, 9]
specI	specI profiling (reference based metagenomics taxonomic profiling [54])
motu	mOTU profiling (taxonomic profiling of metagenomes [55])
minimap2	minimap2 mapper [35]

The bundled databases and modules were chosen because of their wide use at the time that NGLess 1.0 was released. However, they will be updated as appropriate (e.g., when new versions of the underlying databases are available).

## Results and discussion

### Benchmarking

We compared the performance of *NG-meta-profiler* with both MOCAT2 [9] and a pipeline based on calling bwa without preprocessing data and htseq-count [37] for profiling human gut [38] and ocean metagenomes [7]. For this benchmark, eight threads were used (except for the htseq-count software which only supports a single thread).

**Fig. 2:**
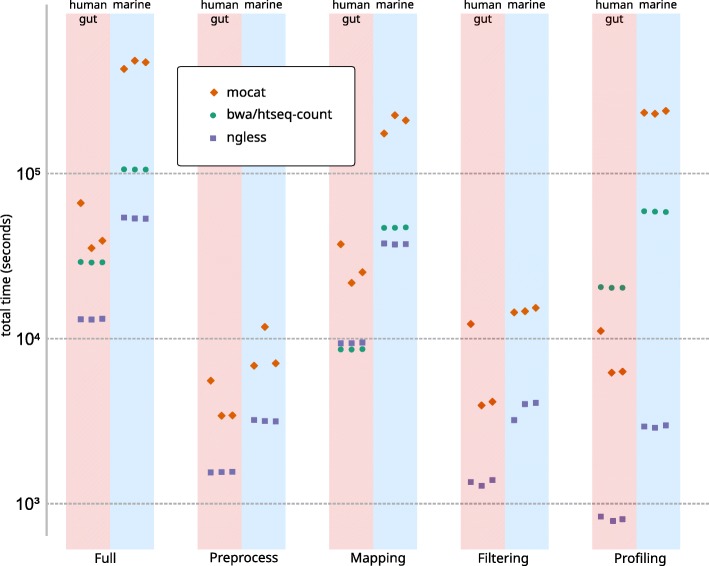
Timing comparison of NGLess and other tools. Three replicates are shown for each tool. The bwa/htseq-count pipeline does not include preprocess and filtering steps

Results are presented in Fig. 2 (see also Additional file 2: Table S1). *NG-meta-profiler* clearly outperforms the other solutions in this task. When compared to MOCAT2, the *NG-meta-profiler* runs 11× faster in the marine benchmark, and 2.6× in the human gut metagenome benchmark. Compared to htseq-count, NGLess is 20–25× faster at building profiles from mapping results (for the marine and the human gut metagenomes, respectively). Even when limited to a single core (for a more direct comparison, as htseq-count does not support multiple cores), NGLess is still 11–16× faster (for the human gut and marine metagenomes, respectively).

The larger ratio in the larger ocean microbial gene catalog (40 million genes) compared to the integrated gene catalog used for profiling the human gut (10 million genes) is evidence that NGLess scales better to very large catalogs.

Note that running the full *NG-meta-profiler* pipeline takes less time than the sum of the individual steps as the pipeline is optimized as a whole (e.g., it avoids generating unnecessary intermediate files).

The three pipelines can perform the same computation, with some minor differences. MOCAT2 defaults to using SOAPAligner [36], while NGLess defaults to using bwa [34], although both tools support both aligners. To test the quality of the results, metagenomes with known taxonomic composition were simulated using abundance and quality distributions derived from real samples. In particular, species distributions were taken from real samples, and reads were simulated from the corresponding genomes. The eggNOG-mapper [23] annotation of the genomes, weighted by the abundance of the corresponding species, was then considered the ground truth. All the three pipelines considered produce results in high agreement with the ground truth (see Table 3 and Additional file 3: Table S2).

**Table 3:** Quality of results based on simulated data

Environment	Tool	Mean	Std. dev.
Human gut	NGLess	88.44	2.07
	MOCAT2	87.32	2.04
	bwa/htseq-count	85.96	1.84
Marine	NGLess	82.26	6.29
	MOCAT2	83.07	6.42
	bwa/htseq-count	82.46	6.32

Shown are the average and std. dev. (over 8 simulations for each environment) of the Spearman rho between the output of each tool and the ground truth

### Pipeline design with NGLess

Using NGLess allows the user to work at a higher level of abstraction than is the case when working directly with traditional command-line tools or generic workflow engines, by using a domain-specific language (DSL). Several tools had already used a DSL to define a computational pipeline. The classical Make tool, which was originally designed for compiling software, but has been used as the basis of a scientific pipeline system [39], uses its own internal language, as do the more modern pipelining tools Snakemake [40] and nextflow [41]. These tools operate by organizing the computation around calls to command-line software. As such, they are fully generic and can be used in a wide range of problems. NGLess trades off this generality to achieve higher usability and performance within its problem domain.

**Fig. 3:**
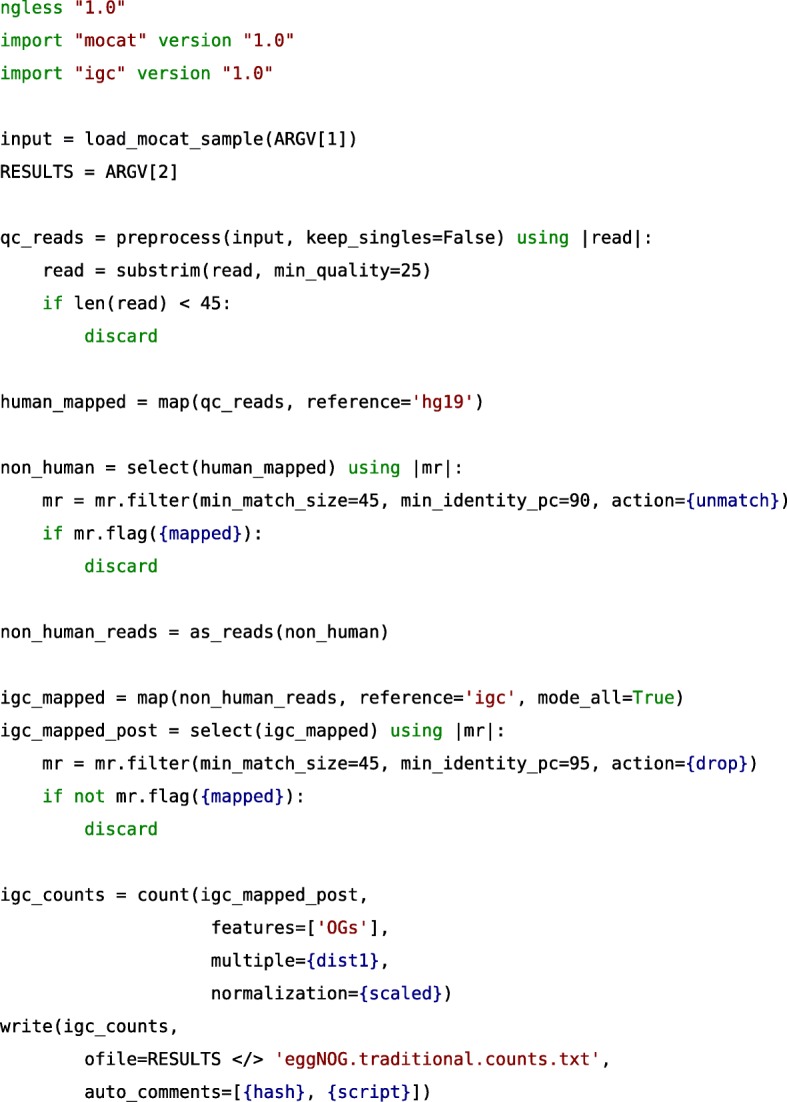
Abridged version of the human profiler, written in the NGLess language

In this section, we illustrate the implementation in NGLess of elements of the human gut profiler (see Figs. 1 and 3) to demonstrate the language and the advantages of using NGLess.

The first element in an NGLess script is the version declaration:
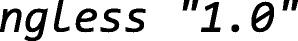


This documents which version of the language the user wants to use. This allows the language to be updated while keeping backward compatibility for older scripts. Already, the current version of the interpreter can run more than one version of the language.

The user can then import helper modules. Specifying versions is required for reproducibility. The first module that needs to be imported is the *mocat* module. This module provides functions to load data that is organized such that each biological sample corresponds to a directory on disk, possibly containing multiple input files. This structure was used in MOCAT/MOCAT2 [8, 9], hence the name of the module. Secondly, we import the *igc* module to be able to use the integrated gene catalog (IGC) of the human gut [25].
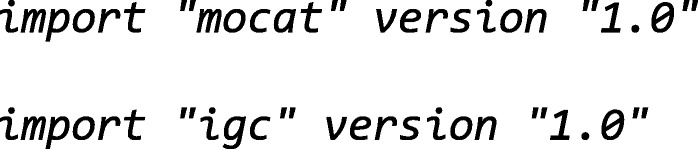


The first step in the pipeline is to load the data. The input directory is given as the first of the command line arguments (which are stored in an array named *ARGV*, following the convention from other programming languages), using the *load_mocat_sample* from the *mocat* module imported above:
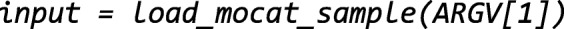


This function can read the FastQ data format in different configurations (single-end, paired-end, multi-lane), which allows transparently handling heterogeneity in read depth and different sequencing strategies.

NGLess is a statically typed language, with automatically inferred types. As such, *input* has type *ShortReadSet*, representing a variable number of paired-end and/or single-end files on disk. To perform quality based trimming and filtering of short reads, the built-in *preprocess* function is used to loop over all the input data:
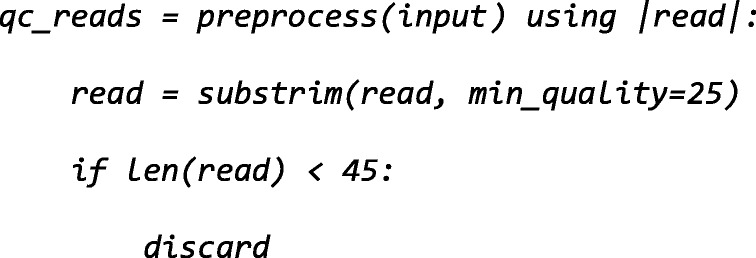


As described above, *substrim* is a built-in NGLess function to trim short-reads based on quality. Alternatively, the user can opt for two additional trimming algorithms, *endtrim* and *smoothtrim*, depending on the sequencing platform and technology used to obtain the raw data (see Additional file 4: Text S1 and the online documentation for details on these functions). While performing the *preprocess* operation, the interpreter will also estimate qualities and generate quality-control plots for both the input and the preprocessed data. These are computed on the data as reads are loaded from disk for preprocessing and before writing it back to disk, so there is no extra input/output (I/O) generated. This behavior would be hard to achieve in standard pipelines where different tasks are often performed by independent software, requiring the same data to be loaded several times.

After preprocessing the short reads, it is necessary to remove reads that match the human genomes as they are not relevant for microbiome studies. For this operation, we will first align the reads against one of the human built-in references, namely *hg19*:
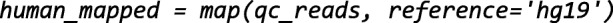


As no aligner is specified explicitly, the default, bwa-mem [34], is used. Independently of which aligner is used, NGLess will ensure that the reference is correctly downloaded and indexed the first time it is used. NGLess will also uncompress and merge multiple input read files before passing the reads to the aligner, while streaming the data so that no additional intermediate files are generated. This is the case not just for the *map* function, but for all tools in NGLess. By using NGLess-based pipelines, the user does not need to be aware of whether these tools support compressed inputs or about subformat issues (e.g., interleaved FastQ files or SAM/BAM distinctions) as these transformations are implicit.

To remove spurious alignments (see Additional file 1: Figure S1), we filter the these aligned reads:
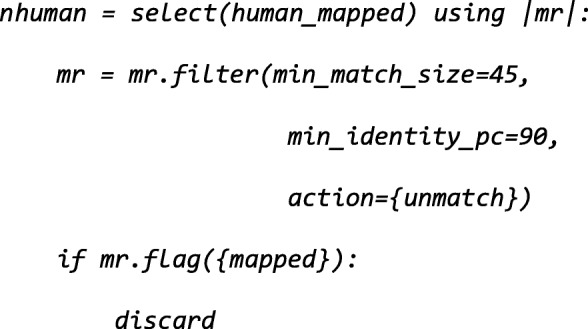


The *nhuman* object is of type *MappedShortReadSet* (the NGLess representation of the information in a SAM/BAM file [42]). In order to extract only the sequences (effectively, converting it back to a set of FastQ files), the *as_reads* function is used:
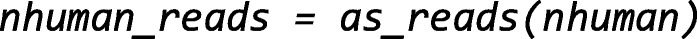


Now, these sequences are mapped to the *igc* reference (the integrated gene catalog, imported earlier). To obtain an abundance table, the *count* function summarizes the mappings:
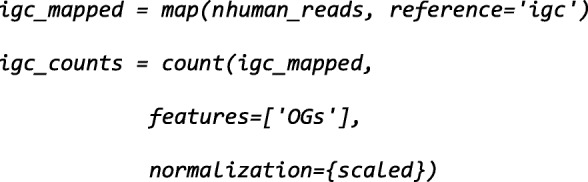


In this case, we quantify eggNOG orthologs groups [43], relying on gene annotations that are part of the *igc* reference. As the annotations are bundled together with the sequence databases, the user does not need to be concerned with the possibility of mismatched identifiers, or even specify the exact paths on disk to the corresponding file.

Finally, the *igc_counts* object can now be saved to disk.
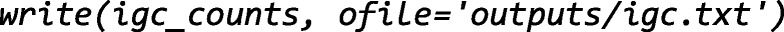


In this case, the file is saved to a subdirectory, namely *outputs/*. If the directory does not exist or if the user does not have permission to write to it, an error will be produced, as would be the case in any other pipeline design. With NGLess, however, the interpreter checks whether this directory exists and can be written to, as early as possible, namely before starting the execution of the pipeline. Thus, in case of error, the user receives a readable error immediately instead of needing to wait for that step of the process to be reached.

### Pipelines developed with NGLess are reproducible

Unlike tools which are based on traditional programming languages, NGLess is designed from the ground up with reproducibility as a goal.

Every pipeline defined with NGLess includes a version declaration and every imported module specifies the particular version which is being imported. Documenting the version of all the tools and dependencies used for a given analysis is considered a best practice [44, 45], but is not always followed. By making it a requirement within the script, NGLess ensures that this best practice is adopted. At the same time, NGLess lowers the necessary effort when compared to having to record the version of all tools and dependencies manually. For example, the versions of samtools [42] and bwa [34] used internally (and shipped with NGLess) are implicitly fixed by specifying the NGLess version. To encourage that credit be given to the original authors, NGLess will print out references for any tools that are used, asking the user to cite them in publications.

Furthermore, although external configuration and command line options may change *how* results are computed (e.g., how many threads to use, where to store temporary files), the results do not depend on any information outside the script. This separation of implementation details from the data processing specification has the added potential of making the resulting code easier to port between systems [46].

### Extensibility and integration into the wider ecosystem of bioinformatics tools

Pipelines defined with NGLess are easily extensible. We encourage users of *NG-meta-profiler* to customize these pipelines to their specific problems and to extend them as desired. Functions can be added to the language based on external software by specifying the interface in a text format and importing it from the main script.

In May 2018, we opened up to the community a repository of external modules (https://github.com/ngless-toolkit/ngless-contrib) where contributions of new integrations are accepted. At the moment, integration of MetaPhlAn2 (a tool for profiling microbial species based on taxa-specific marker genes [47]), mOTUs2 (an updated metagenomics profiler based on univeral marker genes [48]), and salmon (a tool which generates abundance profiles based on k-mers [49]) are available.

Alternatively, NGLess-based analyses can be integrated into larger pipelines. Most existing bioinformatics pipelines are performed with command-line based software. To facilitate integration with existing tools, we provide several tools based on NGLess with a command line interface. In addition, we provide Common Workflow Language (CWL) descriptions of these tools which enable their use as part of CWL workflows [50].

NGLess scripts can (with some limitations) be automatically exported as CWL tools in order to be embedded in larger projects. When passed the *--export-cwl* option, NGLess will output a CWL description of a given script which can then be embedded in a larger CWL-based pipeline.

Finally, NGLess can also be used as an embedded language within Python, a programming language that is widely used for scientific computing [15]. With this interface, NGLess-based pipelines can be defined by Python-based scripts.

### Future developments

In addition to maintenance releases consisting of updates to the databases and their annotations, we plan to keep developing *NG-meta-profiler* and the underlying NGLess framework with a focus on readability and reproducibility. For example, one planned feature is to add support for automatic downloading data from public reference repositories such as the European Nucleotide Archive. Users will then be able to share a small readable script that would access publicly deposited data and process it in a perfectly reproducible fashion. Additionally, several improvements to performance are in progress.

## Conclusions

NGLess puts forward a different approach for defining data analysis pipelines: the use of a domain-specific language for sequence analysis with reproducibility as a design concern.

Using this framework, we developed *NG-meta-profiler* which generates taxonomic and functional profiles from metagenomes based on prebuilt gene catalogs that are provided with the tool and downloaded on first use. When compared with other alternatives, *NG-meta-profiler* performs reference-based functional and taxonomic profiling much faster. Furthermore, this collection of scripts can be easily adapted and extended by the users within the NGLess framework to perform novel functions.

## Availability and requirements

Project name: NG-meta-profiler and NGLess

Project home page: https://ngless.embl.de

Operating system(s): Linux, Mac OS X, and Windows

Programming language: Haskell/NGLess/Python/Bash

Other requirements: None

License: MIT License

Any restrictions to use by non-academics: None

## Additional files

Additional file 1: **Figure S1.** Fraction of spurious hits to the human genome that is incorrectly kept as a function of the minimum size of the alignment used to consider it a valid alignment. The benchmark simulated dataset was aligned to the human genome. As in this simulated dataset there should be no human reads, any alignment was considered spurious. (PDF 52 kb)

Additional file 2: **Table S1.** Complete timing information for benchmark on real data for NGLess, MOCAT2, and bwa/htseq-count. (XLSX 11 kb)

Additional file 3: **Table S2.** Full results on the synthetic benchmark, shown are the Spearman correlation values between the estimated OG abundances and the distribution being simulated. (XLSX 6 kb)

Additional file 4: Text S1. The NGLess Language and Standard Library (PDF 129 kb)
