# Supplementary material for: NG-meta-profiler: fast processing of metagenomes using NGLess, a domain-specific language
Source: Microbiome. 2019 Jun 3;7:84. doi: 10.1186/s40168-019-0684-8 (PMC6547473; doi:10.1186/s40168-019-0684-8)
Supplement: Supplementary file 1 — Figure S1. Fraction of spurious hits to the human genome that is incorrectly kept as a function of the minimum size of the alignment used to consider it a valid alignment. The benchmark simulated dataset was aligned to the human genome. As in this simulated dataset there should be no human reads, any alignment was considered spurious. (PDF 52 kb) [file 40168_2019_684_MOESM1_ESM.pdf]

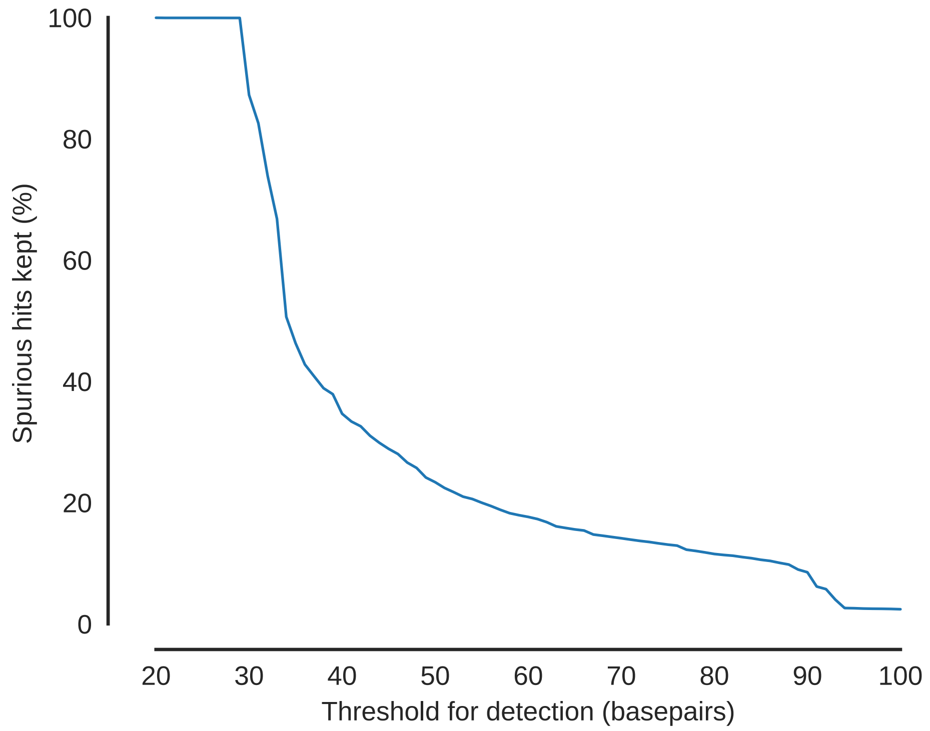

Supplemental Figure 1: Fraction of spurious hits to the human genome that is incorrectly kept as a function of the minimum size of the alignment used to consider it a valid alignment. The benchmark simulated dataset was aligned to the human genome. As in this simulated dataset there should be no human reads, any alignment was considered spurious.
