# Supplementary material for: NG-meta-profiler: fast processing of metagenomes using NGLess, a domain-specific language
Source: Microbiome. 2019 Jun 3;7:84. doi: 10.1186/s40168-019-0684-8 (PMC6547473; doi:10.1186/s40168-019-0684-8)
Supplement: Supplementary file 4 — Text S1. The NGLess Language and Standard Library (PDF 129 kb) [file 40168_2019_684_MOESM4_ESM.pdf]

Supplemental File 1: The NGLess Language and Standard Library

Luis Pedro Coelho
Renato Alves
Paulo Monteiro
Jaime Huerta-Cepas
Ana Teresa Freitas
Peer Bork
March 21, 2019

NGLess Language

This document describes the NGLess language. Please see http://ngless.embl.de for an up-to-date version.

Tokenization

Tokenization follows the standard C-family rules. A word is anything that matches `[A-Za-z_][A-Za-z_0-9]*`. The language is case-sensitive. All files are assumed to be in UTF-8.

Both LF and CRLF are accepted as line endings (Unix-style LF is preferred).

A semicolon (;) can be used as an alternative to a new line. Any spaces (and only space characters) following a semicolon are ignored. *This feature is intended for inline scripts at the command line (passed with the `-e` option), its use for scripts is heavily discouraged and may trigger an error in the future.*

Script-style (# to EOL), C-style (/* to */) and C++-style (// to EOL) comments are all recognised. Comments are effectively removed prior to any further parsing as are empty lines.

Strings are denoted with single or double quotes and standard backslashed escapes apply (\n for newline, ...).

A symbol is denoted as a token surrounded by curly braces (e.g., `{symbol}` or `{gene}`).

Integers are specified as decimals `[0-9]+` or as hexadecimals `0x[0-9a-fA-F]+`.

Version declaration

The first line (ignoring comments and empty lines) of an NGLess file MUST be a version declaration:

```
ngless "1.0"
```

Module Import Statements

Following the version statement, optional import statements are allowed, using the syntax import "<MODULE>" version "<VERSION>". For example:

```
import "batch" version "1.0"
```

This statement indicates that the `batch` module, version `1.0` should be used in this script. Module versions are independent of NGLess versions.

Only a predefined set of modules can be imported (these are shipped with NGLess). To import user-written modules, the user MUST use the *local import* statement, e.g.:

```
local import "batch" version "1.0"
```

Import statements MUST immediately follow the version declaration

Blocks

Blocks are defined by indentation in multiples of 4 spaces. To avoid confusion, TAB characters are not allowed.

Blocks are used for conditionals and `using` statements.

Data types

NGless supports the following basic types:

- String
- Integer
- Double
- Bool
- Symbol
- Filename
- Shortread
- Shortreadset
- Mappedread
- Mappedreadset

In addition, it supports the composite type List of X where X is a basic type. Lists are built with square brackets (e.g., [1,2,3]). All elements of a list must have the same data type.

String

A string can start with either a quote `(U+0022, ")` or a single quote `(U+0027,')` or and end with the same character. They can contain any number of characters.

Special sequences start with `\`. Standard backslashed escapes can be used as `LF` and `CR` (`\n` and `\r` respectively), quotation marks (`\'`) or slash (`\\`).

Integer

Integers are specified as decimals `[0-9]+` or as hexadecimals `0x[0-9a-fA-F]+`. The prefix `-` denotes a negative number.

Double

Doubles are specified as decimals `[0-9]+` with the decimal point serving as a separator. The prefix `-` denotes a negative number.

Doubles and Integers are considered numeric types.

Boolean

The two boolean constants are `True` and `False` (which can also be written `true` or `false`).

Symbol

A symbol is denoted as a token surrounded by curly braces (e.g., `{symbol}` or `{drop}`). Symbols are used as function arguments to indicate that there is only a limited set of allowed values for that argument. Additionally, unlike Strings, no operations can be performed with Symbols.

Variables

NGless is a statically typed language and variables are typed. Types are automatically inferred from context.

Assignment is performed with = operator:

```
variable = value
```

A variable that is all uppercase is a constant and can only be assigned to once.

Operators

Unary

The operator `(-)` returns the symmetric of its numeric argument.

The operator `len` returns the length of a ShortRead.

The operator `not` negates its boolean argument

Binary

All operators can only be applied to numeric types. Mixing integers and doubles returns a double. The following binary operators are used for arithmetic:

```
+ - < > >= <= == !=
```

The + operator can also perform concatenation of String objects.

The </> operator is used to concatenate two Strings while also adding a '/' character between them. This is useful for concatenating file paths.

Indexing

Can be used to access only one element or a range of elements in a ShortRead. To access one element, is required an identifier followed by an expression between brackets. (e.g, x[10]).

To obtain a range, is required an identifier and two expressions separated by a ':' and between brackets. Example:

- `x[:]` - from position 0 until length of variable x
- `x[10:]` - from position 10 until length of variable x
- `x[:10]` - from position 0 until 10

Conditionals

Conditionals work as in Python. For example:

```
if 5 > 10:
   val = 10
else:
   val = 20
```

Functions

Functions are called with parentheses:

```
result = f(arg, arg1=2)
```

Functions have a single positional parameter, all other must be given by name:

```
unique(reads, max_copies=2)
```

The exception is constructs which take a block: they take a single positional parameter and a block. The block is passed using the `using` keyword:

```
reads = preprocess(reads) using |read|:
    block
    ...
```

The |read| syntax defines an unnamed (lambda) function, which takes a variable called read. The function body is the following block.

There is no possibility of defining new functions within the language. Only built-in functions or those added by modules can be used.

Methods

Methods are called using the syntax object . methodName (<ARGS>). As with functions, one argument may be unnamed, all others must be passed by name.

Grammar

This is the extended Backus-Naur form grammar for the NGLess language (using the ISO 14977 conventions). Briefly, the comma (,) is used for concatenation, [x] denotes *optional*, and {x} denotes *zero or more of x*.

```
string = ? a quoted string, produced by the tokenizer ? ;
word = ? a word produced by the tokenizer ? ;

eol =
    ';'
    | '\n' {'\n'}
    ;


ngless = [header], body;

header = {eol}, ngless_version, {eol}, {import}, {eol}

ngless_version = "ngless", string, eol ;

import = ["local"],  "import", string, "version", string, eol ;

body = {expression, eol} ;

expression =
            conditional
            | "discard"
            | "continue"
            | assignment
            | innerexpression
            ;

innerexpression = left_expression, binop, innerexpression
                    | left_expression
                    ;

left_expression =  uoperator
```

```
                    | method_call
                    | indexexpr
                    | base_expression
                    ;

base_expression = pexpression
                   | funccall
                   | listexpr
                   | constant
                   | variable
                   ;

pexpression = '(', innerexpression, ')' ;

constant =
        "true"
        | "True"
        | "false"
        | "False"
        | double
        | integer
        | symbol
        ;

double = integer, '.', integer ;
integer = digit, {digit} ;
digit = '0' | '1' | '2' | '3' | '4' | '5' | '6' | '7' | '8' | '9'
;
symbol = '{', word, '}' ;


indentation = ' ', {' '} ;
binop = '+' | '-' | '*' | "!=" | "==" | "</>" | "<=" | "<" | ">="
| ">" | "+" | "-" ;

uoperator =
        lenop
        | unary_minus
        | not_expr
        ;

lenop = "len", '(', expression, ')'
unary_minus = '-', base_expression ;
not_expr = "not", innerexpression ;

funccall = paired
        | word,  '(', innerexpression, kwargs, ')', [ funcblock ]
        ;
```

```
(* paired is a special-case function with two arguments *)
paired = "paired", '(', innerexpression, ',', innerexpression,
kwargs ;

funcblock = "using", '|', [ variablelist ], '|', ':', block ;


kwargs = {',', variable, '=', innerexpression} ;

assignment = variable, '=', expression ;

method_call = base_expression, '.', word, '(', [ method_args ],
')';
method_args =
        innerexpression, kwargs
        | variable, '=', innerexpression, kwargs
        ; (* note that kwargs is defined as starting with a comma
*)

indexexpr = base_expression, '[', [ indexing ], ']' ;

indexing = [ innerexpression  ], ':', [ innerexpression ] ;

listexpr = '[', [ list_contents ] , ']' ;
list_contents = innerexpression, {',', innerexpression } ;

conditional = "if",  innerexpression, ':',  block,
[ elseblock ] ;
elseblock = "else", ':', block ;
block = eol, indentation, expression, eol, {indentation,
expression, eol} ;

variablelist = variable, {',', variable} ;
variable = word ;
```

NGLess Builtin Functions

fastq

Function to load a FastQ file:

```
in = fastq('input.fq')
```

Argument:

String

Return:

ReadSet

Arguments by value:

Name	Type	Required	Default Value
encoding	Symbol ({auto}, {33}, {64}, {sanger}, {solexa})	no	{auto}

Possible values for `encoding` are:

- `{sanger}` or `{33}` assumes that the file is encoded using sanger format. This is appropriate for newer Illumina outputs.
- `{solexa}` or `{64}` assumes that the file is encoded with a 64 offset. This is used for older Illumina/Solexa machines.
- `{auto}`: use auto detection. This is the default.

When loading a data set, quality control is carried out and statistics can be visualised in a graphical user interface (GUI). Statistics calculated are:

- percentage of guanine and cytosine (%GC)
- number of sequences
- minimum/maximum sequence length
- mean, median, lower quartile and upper quality quartile for each sequence position

If not specified, the encoding is guessed from the file.

Gzip and bzip2 compressed files are transparently supported (determined by file extension, `.gz` and `.bz2` for gzip and bzip2 respectively).

paired

Function to load a paired-end sample, from two FastQ files:

```
in = paired('input.1.fq', 'input.2.fq', singles='input.3.fq')
```

`paired()` is an exceptional function which takes **two** unnamed arguments, specifying the two read files (first mate and second mate) and an optional `singles` file (which contains unpaired reads).

Argument:

String, String

Return:

ReadSet

Arguments by value:

Name	Type	Required	Default Value
encoding	Symbol ({auto}, {33}, {64}, {sanger}, {solexa})	no	{auto}
singles	String	no	

The `encoding` argument has the same meaning as for the `fastq()` function:

- `{sanger}` or `{33}` assumes that the file is encoded using sanger format. This is appropriate for newer Illumina outputs.
- `{solexa}` or `{64}` assumes that the file is encoded with a 64 offset. This is used for older Illumina/Solexa machines.
- `{auto}`: use auto detection. This is the default.

samfile

Loads a SAM file:

```
s = samfile('input.sam')
```

This function takes no keyword arguments. BAM files are also supported (determined by the filename), as are `sam.gz` files.

Returns

MappedReadSet

Arguments by value:

Name	Type	Required	Default Value
name	String	no	
header	String	no	

Note: The `header` argument was added in version 0.7

- The `name` argument names the group (for `count()`, for example).
- The `headers` argument can be used if the SAM headers are kept in a separate file.

qcstats

Note: This functionality was not available prior to 0.6

Returns the auto-computed statistics:

```
write(qcstats({fastq}), ofile='fqstats.txt')
```

Returns

CountsTable

Argument

`{fastq}`: FastQ statistics `{mapping}`: Mapping statistics

countfile

Loads a TSV file:

```
c = countfile('table.tsv')
```

This function takes no keyword arguments. If the filename ends with ".gz", it is assumed to be a gzipped file.

Returns

CountTable

as_reads

Converts from a `MappedReadSet` to a `ReadSet`:

```
reads = as_reads(samfile('input.sam'))
```

unique

Function that given a set of reads, returns another which only retains a set number of copies of each read (if there are any duplicates). An example:

```
input = unique(input, max_copies=3)
```

Argument:

ReadSet

Return:

ReadSet

Arguments by value:

Name	Type	Required	Default Value
max_copies	Integer	no	2

The optional argument **max_copies** allows to define the number of tolerated copies (default: 2).

Two short reads with the same nucleotide sequence are considered copies, independently of quality and identifiers.

This function is currently limited to single-end samples.

preprocess

This function executes the given block for each read in the ReadSet. Unless the read is **discarded**, it is transferred (after transformations) to the output. For example:

```
inputs = preprocess(inputs) using |read|:
    read = read[3:]
```

Argument:

ReadSet

Return:

ReadSet

Arguments by value:

Name	Type	Required	Default Value
keep_singles	bool	no	true

When a paired-end input is being preprocessed in single-mode (i.e., each mate is preprocessed independently, it can happen that on eof the mates is discarded, while the other is kept). The default is to collect these into the singles pile. If `keep_singles` if false, however, they are discarded.

This function also performs quality control on its output.

map

The function map, maps a ReadSet to reference. For example:

```
mapped = map(input, reference='sacCer3')
mapped = map(input, fafile='ref.fa')
```

Argument:

ReadSet

Return:

MappedReadSet

Arguments by value:

Name	Type	Required	Default Value

reference	String	no
fafile	String	no
block_size_megabases	Integer	no
mode_all	Bool	no

The user must provide either a path to a FASTA file in the `fafile` argument or the name of a builtin reference using the `reference` argument. The `fafile` argument supports search path expansion.

A list of datasets provided by NGLess can be found at Organisms.

To use any of these, pass in the name as the reference value:

```
mapped_hg19 = map(input, reference='hg19')
```

NGLess does not ship with any of these datasets, but they are downloaded lazily: i.e., the first time you use them, NGLess will download and cache them. NGLess will also index any database used the first time it is used.

The option `block_size_megabases` turns on low memory mode (see the corresponding section in the mapping documentation)

The option `mode_all=True` can be passed to include all alignments of both single and paired-end reads in the output SAM/BAM.

mapstats

Computes some basic statistics from a set of mapped reads (number of reads, number mapped, number uniquely mapped).

Argument

MappedReadSet

Return

CountTable

select

select filters a MappedReadSet. For example:

```
mapped = select(mapped, keep_if=[{mapped}])
```

Argument:

MappedReadSet

Return:

MappedReadSet

Arguments by value:

Name	Type	Required	Default Value
keep_if	[Symbol]	no	
drop_if	[Symbol]	no	
paired	Bool	no	true

At least one of `keep_if` or `drop_if` should be passed, but not both. They accept the following symbols:

- `{mapped}`: the read mapped
- `{unmapped}`: the read did not map
- `{unique}`: the read mapped to a unique location

If `keep_if` is used, then reads are kept if they pass **all the conditions**. If `drop_if` they are discarded if they fail to **any condition**.

By default, `select` operates on a paired-end read as a whole. If `paired=False` is passed, however, then link between the two mates is not considered and each read is processed independently.

count

Given a file with aligned sequencing reads (ReadSet), `count()` will produce a counts table depending on the arguments passed. For example:

```
counts = count(mapped, min=2, mode={union}, multiple={dist1})
```

Argument:

MappedReadSet

Return:

CountTable

Arguments by value:

Name	Type	Required	Default value
gff_file	String	no*	
functional_map	String	no*	
features	[String]	no	'gene'

subfeatures	[String]	no	
mode	Symbol	no	{union}
multiple	Symbol	no	{dist1}
strand	Bool	no	false
normalization	Symbol	no	{raw}
include_minus1	Bool	no	true
min	Integer	no	0
discard_zeros	Bool	no	false
reference	String	no	""

If the features to count are `['seqname']`, then each read will be assigned to the name of reference it matched and only an input set of mapped reads is necessary. For other features, you will need extra information. This can be passed using the `gff_file` or `functional_map` arguments. If you had previously used a `reference` argument for the `map()` function, then you can also leave this argument empty and NGLess will use the corresponding annotation file.

The `gff_file` and `functional_map` arguments support search path expansion.

`features`: which features to count. If a GFF file is used, this refers to the "features" field.

`subfeatures`: this is useful in GFF-mode as the same feature can encode multiple attributes (or, in NGLess parlance, "subfeatures"). By default, NGLess will look for the `"ID"` or `"gene_id"` attributes.

`mode` indicates how to handle reads that (partially) overlap one or more features. Possible values for `mode` are `{union}`, `{intersection_non_empty}` and `{intersection_strict}` (default: `{union}`). For every position of a mapped read, collect all features into a set. These sets of features are then handled in different modes.

- `{union}` the union of all the sets. A read is counted for every feature it overlaps.
- `{intersection_non_empty}` the intersection of all non-empty sets. A read is only counted for features it exclusively overlaps, even if partially.
- `{intersection_strict}` the intersection of all the sets. A read is only counted if the entire read overlaps the same feature(s).

Consider the following illustration of the effect of different `mode` options:

```
Reference *************************
Feature A      =======
Feature B            ===========
Feature C                 ========
Read_1       -----
Read_2             -----
Read_3                    -----
Position     12345 12345  12345

Read position          1    2    3    4    5
Read_1 feature sets    -    -    A    A    A
Read_2 feature sets    A    A  A,B    B    B
Read_3 feature sets  B,C  B,C  B,C  B,C  B,C

           union  intersection_non_empty  intersection_strict
Read_1        A                       A                    -
Read_2     A & B                      -                    -
Read_3     B & C                  B & C                B & C
```

How to handle multiple mappers (inserts which have more than one "hit" in the reference) is defined by the `multiple` argument:

- `{unique_only}`: only use uniquely mapped inserts
- `{all1}`: count all hits separately. An insert mapping to 4 locations adds 1 to each location
- `{1overN}`: fractionally distribute multiple mappers. An insert mapping to 4 locations adds 0.25 to each location
- `{dist1}`: distribute multiple reads based on uniquely mapped reads. An insert mapping to 4 locations adds to these in proportion to how uniquely mapped inserts are distributed among these 4 locations.

Argument `strand` represents whether the data are from a strand-specific (default is `false`). When the data is not strand-specific, a read is always overlapping with a feature independently of whether maps to the same or the opposite strand. For strand-specific data, the read has to be mapped to the same strand as the feature.

`min` defines the minimum amount of overlaps a given feature must have, at least, to be kept (default: 0, i.e., keep all counts). If you just want to discard features that are exactly zero, you should set the `discard_zeros` argument to True.

`normalization` specifies if and how to normalize to take into account feature size:

- `{raw}` (default) is no normalization
- `{normed}` is the result of the `{raw}` mode divided by the size of the feature
- `{scaled}` is the result of the `{normed}` mode scaled up so that the total number of counts is identical to the `{raw}` (within rounding error)

Unmapped inserts are included in the output if `{include_minus1}` is true (default: `False`).

Note: Before version 0.6, the default was to **not** include the -1 fraction.

substrim

Given a read finds the longest substring, such that all bases are of at least the given quality. The name is a constraction of "substring trim". For example:

```
read = substrim(read, min_quality=25)
```

Argument:

ShortRead

Return:

ShortRead

Arguments

Name	Type	Required	Default Value
min_quality	Integer	yes	

`min_quality` parameter defines the minimum quality accepted for the sub-sequence.

endstrim

Given a read, trim from both ends (5' and 3') all bases below a minimal quality. For example:

```
read = endstrim(read, min_quality=25)
```

Argument:

ShortRead

Return:

ShortRead

Arguments

Name	Type	Required	Default Value
min_quality	Integer	yes	

`min_quality` parameter defines the minimum quality value.

smoothtrim

This trims with the same algorithm as `substrim` but uses a sliding window to average base qualities. For example:

```
read = smoothtrim(read, min_quality=15, window=4)
```

Quality values of bases at the edges of each read are repeated to allow averaging with quality centered on each base. For instance a read:

```
Sequence   A  T  C  G    with a window     A  A  T  C  G  G
Quality   28 25 14 12  of size 3 becomes  28 28 25 14 12 12
```

and is smoothed:

```
Seq         A  A  T  C  G  G   smoothed quality   A  T  C  G
Qual       28 28 25 14 12 12         --->        27 22 17 13
Windows     |-----|            (28 + 28 + 25) / 3 = 27     ^
 ...           |-----|         (28 + 25 + 14) / 3 = 22     |
                  |-----|      (25 + 14 + 12) / 3 = 17     !
                     |-----|   (14 + 12 + 12) / 3 = 13 ----+
```

at which point `substrim` is applied for trimming.

Quality scores are returned to their original value after trimming.

Argument

ShortRead

Return

ShortRead

Arguments

Name	Type	Required	Default Value
min_quality	Integer	yes	
window	Integer	yes	

`min_quality` parameter defines the minimum quality value, whilst `window` defines the size of the window over which to smooth the qualities.

write

Writes an object to disk.

ReadSet

Argument:

Any

Return:

Void

Arguments by value:

Name	Type	Required	Default Value
ofile	String	yes	
format	String	no	

The argument `ofile` is where to write the content.

The output format is typically determined from the `ofile` extension, but the `format` argument overrides this. Supported formats:

- CountsTable: `{tsv}` (default) or `{csv}`: use TAB or COMMA as a delimiter
- MappedReadSet: `{sam}` (default) or `{bam}`
- ReadSet: FastQ format, optionally compressed (depending on the extension).

print

Print function allows to print a NGLessObject to IO.

Argument:

NGLessObject

Return:

Void

Arguments by value:

none

readlines

Reads a text file and returns a list with all the strings in the file

Argumment

string: the filename

Example

`readlines` is useful in combination with the parallel module, where you can then use the `lock1` function to process a large set of inputs:

```
sample = lock1(readlines('samplelist.txt'))
```

assemble

assemble

Implementation

assemble() uses the MEGAHIT assembler.

Arguments

ReadSet

Returns

string : generated file

orf_find

orf_find finds open reading frames (ORFs) in a sequence set:

```
contigs = assemble(input)
orfs = select(contigs, is_metagenome=True)
```

Argument:

SequenceSet

Return:

SequenceSet

Arguments by value:

Name	Type	Required	Default Value
is_metagenome	Bool	yes	
coords_out	FilePath	no	
prots_out	FilePath	no	

Implementation

NGLess uses Prodigal as the underlying gene finder.

Methods

Methods are invoked using an object-oriented syntax. For example:

```
mapped = select(mapped) using |mr|:
    mr = mr.pe_filter()
```

They can also take arguments

```
mapped = select(mapped) using |mr|:
    mr = mr.filter(min_match_size=30)
```

Short reads

Short reads have the following methods:

- `avg_quality()`: the average quality (as a double)
- `fraction_at_least(q)`: the fraction of bases of quality greater or equal to `q`
- `n_to_zero_quality()`: transform the quality scores so that any `N` (or `n`) bases in the sequence get a quality of zero.

Mapped reads

Mapped reads contain several methods. *None of these methods changes its argument, they return new values.* The typical approach is to reassign the result to the same variable as before (see examples above).

- `pe_filter`: only matches where both mates match are kept.
- `flag`: Takes one of `{mapped}` or `{unmapped}` and returns true if the reads were mapped (in a paired-end setting, a read is considered mapped if at least one of the mates mapped).
- `some_match`: Takes a reference name and returns True if the read mapped to that reference name.
- `allbest`: eliminates matches that are not as good as the best. For NGLess, the number of errors (given by the `NM` field) divided by the length of the longest match is the fractional distance of a match. Thus, a match with 3 errors over 100 bp is considered better than a match with 0 errors over 90bps.

filter

`filter` takes a mapped read and returns a mapped read according to several criteria:

- `min_match_size`: minimum match size
- `min_identity_pc`: minimum percent identity (considered over the matching location, trimming on the left and right are excluded).
- `max_trim`: maximum number of bases trimmed off the ends. Use 0 to specify only global matches.

If more than one test is specified, then they are combined with the AND operation (i.e., all conditions have to be fulfilled for the test to be true).

The default is to discard mappings that do not pass the test, but it can be changed with the `action` argument, which must be one of `{drop}` (default: the read is excluded from the output), or `{unmatch}` (the read is changed so that it no longer reports matching).

You can pass the flag `reverse` (i.e., `reverse=True`) to reverse the sign of the test.

NGLess Constants

In NGLess, any variable written in uppercase is a constant, i.e., can only be assigned to once. In addition, there are builtin constants defined by NGLess.

Built in constants

- ARGV

This is string array which contains the arguments passed to the script

- STDIN

Use in place of a filename to read from standard input

- STDOUT

Use in place of a filename to write to standard output

For example:

```
ngless '1.0'

input = samfile(STDIN)
input = select(input) using |mr|:
    if mr.flag({mapped}):
        discard
write(input, ofile=STDOUT, format={bam})
```

This file reads a sam stream from stdin, filters it (using the `select` call) and writes to standard output in bam format.

Standard library

Parallel module

This module allows you to run several parallel computations. It provides two functions: `lock1` and `collect`.

`lock1 :: [string] -> string` takes a list of strings and returns a single element. It uses the filesystem to obtain a lock file so that if multiple processes are running at once, each one will return a different element. NGLess also marks results as *finished* once you have run a script to completion.

The intended usage is that you simply run as many processes as inputs that you have and NGLess will figure everything out.

For example

```
ngless "0.6"
import "parallel" version "0.6"

samples = ['Sample1', 'Sample2', 'Sample3']
current = lock1(samples)
```

Now, current will be one of `'Sample1'`, `'Sample2'`, or `'Sample3'`. You can use this to find your input data:

```
input = paired("data/" + current + ".1.fq.gz", "data/" + current
+ ".2.fq.gz")
```

Often, it's a good idea to combine `lock1` with `readlines` (a function which returns the contents of all the non-empty lines in a file as a list of strings):

```
samples = readlines('samples.txt')
current = lock1(samples)
input = paired("data/" + current + ".1.fq.gz", "data/" + current
+ ".2.fq.gz")
```

You now use `input` as in any other NGLess script:

```
mapped = map(input, reference='hg19')
write(input, ofile='outputs/'+current+ '.bam')
counts = count(mapped)
write(counts, ofile='outputs/'+current+ '.txt')
```

This will result in both BAM files and counts being written to the `outputs/` directory. The module also adds the `collect` function which can paste all the counts together into a single table, for convenience:

```
collect(
    counts,
    current=current,
    allneeded=samples,
    ofile='outputs/counts.txt.gz')
```

Now, only when all the samples in the `allneeded` argument have been processed, does NGLess collect all the results into a single table.

Full "parallel" example

```
ngless "0.8"
import "parallel" version "0.6"

sample = lock1(readlines('input.txt'))
input = fastq(sample)
mapped = map(input, reference='hg19')
collect(count(mapped, features=['seqname']),
    current=sample,
    allneeded=readlines('input.txt'),
    ofile='output.tsv')
```

Now, you can run multiple `ngless` jobs in parallel and each will work on a different line of `input.txt`.

Parallel internals

Normally this should be invisible to you, but if you are curious or want to debug an issue, here are the gory details:

The function `lock1()` will create a lock file in a sub-directory of `ngless-locks`. This directory will be named by the hash value of the script. Thus, any change to the script will force all data to be recomputed. This can lead to over-computation but it ensures that you will always have the most up to date results (NGLess' first priority is correctness, performance is important, but not at the risk of correctness). Similarly, `collect()` will use hashed values which encode both the script and the position within the script (so that if you have more than one `collect()` call, they will not clash).

Lock files have their modification times updated once every 10 minutes while NGLess is running. This allows the programme to easily identify stale files. The software is very conservative, but any lock file with a modification time older than one hour is considered stale and removed. Note that because NGLess will write always create its outputs atomically, the worse that can happen from mis-identifying a stale lock (for example, you had a compute node which lost network connectivity, but it comes back online after an hour and resumes processing) is that extra computation is wasted, **the processes will never interfere in a way that you get erroneous results**.

Samtools module

This module exposes the samtools sorting functionality through the `samtools_sort` function.

```
ngless '0.0'
import "samtools" version "0.0"
to_sort = samfile('input.bam')
sorted = samtools_sort(to_sort)
write(sorted, ofile='input.sorted.bam')
```

`samtools_sort :: mappedreadset -> mappedreadset` returns a sorted version of the dataset.

Internally, this function calls NGLess' version of samtools while respecting your settings for the use of threads and temporary disk space. When combined with other functionality, NGLess can also often stream data into/from samtools instead of relying on intermediate files (these optimizations should not change the visible behaviour, only make the computation faster).

Mocat module

```
import "mocat" version "0.6"
```

This is a MOCAT compatibility layer to make it easier to adapt projects from MOCAT to NGLess.

Functions

`load_mocat_sample :: string -> readset` this function takes a directory name and returns a set of reads by scanning the directory for (compressed) FastQ files. This is slightly more flexible than MOCAT2 as it also accepts files with the extension `fastq` or `fastq.gz` as well as _1 and _2 to indicate the two mate files.

`coord_file_to_gtf :: string -> string` this function takes a MOCAT-style `.coord`, converts it internally to a GTF file and returns it.

Example usage:

```
ngless "0.6"
import "mocat" version "0.6"

sample = load_mocat_sample('Sample1')
mapped = map(sampled, fafile='data/catalog.padded.fna')
write(count(mapped,
gff_file=coord_file_to_gtf('data/catalog.padded.coord')),
    ofile='counts.txt')
```

This module can be combined with the parallel module (see above) to obtain a very smooth upgrade from MOCAT to NGLess.

Available Reference Genomes

NGLess provides builtin support for the most widely used model organisms (human, mouse, yeast, C. elegans, ...; see the full table below). This makes it easier to use the tool when using these organisms as some knowledge is already built in.

Genome references available

NGLess provides archives containing data sets of organisms. Is also provided gene annotations that provide information about protein-coding and non-coding genes, splice variants, cDNA and protein sequences, non-coding RNAs.

The following table lists the genomes provided by default:

Name	Description	Assembly	Ensembl
bosTau4	bos_taurus	UMD3.1	75
ce10	caenorhabditis_elegans	WBcel235	75
canFam3	canis_familiaris	CanFam3.1	75
dm6	drosophila_melanogaster	BDGP6	90
dm5	drosophila_melanogaster	BDGP5	75
gg5	gallus_gallus	Gallus_gallus-5.0	90
gg4	gallus_gallus	GalGal4	75
hg38.p10	homo_sapiens	GRCh38.p10	90
hg38.p7	homo_sapiens	GRCh38.p7	85
hg19	homo_sapiens	GRCh37	75
mm10.p5	mus_musculus	GRCm38.p5	90
mm10.p2	mus_musculus	GRCm38.p2	75
rn6	rattus_norvegicus	Rnor_6.0	90
rn5	rattus_norvegicus	Rnor_5.0	75
sacCer3	saccharomyces_cerevisiae	R64-1-1	75
susScr11	sus_scrofa	Sscrofa11.1	90

These archives are all created using versions 75, 85 and 90 of Ensembl.
